# Non-concealed placebo treatment for menopausal hot flushes: Study protocol of a randomized-controlled trial

**DOI:** 10.1186/s13063-019-3575-1

**Published:** 2019-08-16

**Authors:** Yiqi Pan, Ramona Meister, Bernd Löwe, Anne Winkelmann, Ted J. Kaptchuk, Kai J. Buhling, Yvonne Nestoriuc

**Affiliations:** 1 Department of Psychosomatic Medicine and Psychotherapy, University Medical Center Hamburg-Eppendorf, Martinistraße 52, 20246 Hamburg, Germany; 2 Department of Medical Psychology, University Medical Center Hamburg-Eppendorf, Martinistraße 52, 20246 Hamburg, Germany; 3 Program in Placebo Studies and the Therapeutic Encounter (PiPS), Beth Israel Deaconess Medical Center, Harvard Medical School, 330 Brookline Avenue, Boston, MA 02215 USA; 4 Clinic for Gynecology, Department of Gynecological Endocrinology, University Medical Center Hamburg-Eppendorf, Martinistraße 52, 20246 Hamburg, Germany; 5 Clinical Psychology, Helmut-Schmidt-University / University of the Federal Armed Forces Hamburg, Holstenhofweg 85, 22043 Hamburg, Germany

**Keywords:** Hot flushes, Hot flashes, Menopause, Open-label placebo, Placebo effects, Non-hormonal treatment

## Abstract

**Background:**

Beneficial effects of placebos are high in double-blind hot flush trials. Studies in various conditions suggest that honestly prescribed placebos may elicit symptom improvement.

**Objective:**

To determine whether open label placebo (OLP) treatment is efficacious in alleviating hot flushes among peri- and postmenopausal women.

**Methods/design:**

In this assessor-blinded, randomized-controlled trial, *n* = 100 women experiencing five or more daily hot flushes of at least moderate severity and bothersomeness are assigned 1:1 to a 4-week OLP treatment or no treatment. To explore the duration and maintenance of placebo effects, the OLP group is randomized a second time to either discontinue or continue the OLP treatment for another 4 weeks. All participants receive a briefing about placebo effects and study visits at baseline, post-treatment (4 weeks), and follow-up (8 weeks, OLP group only). Qualitative interviews about subjective experiences with the OLP treatment are conducted.

Primary outcomes are differences between the OLP and the no-treatment group in the hot flush composite score (frequency × severity), and bothersomeness of hot flushes as assessed with the Hot Flush Rating Scale at post-treatment. Secondary outcomes include hot flush frequency, health-related quality of life, global improvement, and the number of responders at post-treatment. Data are analyzed by fitting (generalized) linear mixed models. An exploratory analysis of maintenance and duration is performed including follow-up data.

**Discussion:**

This trial will contribute to the evaluation of OLP treatments in clinical practice and further our understanding about the magnitude of placebo effects in hot flush treatments.

**Trial registration:**

Clinicaltrials.gov, NCT03838523. Retrospectively registered on February 12th, 2019. The first patient was enrolled on October 10th, 2018.

## Background

Hot flushes are the most common symptoms related to menopause [1]. Around 16–74% of women worldwide experience hot flushes, persisting on average for 7.4 years, with some women reporting sustained symptoms for up to 14 years [2]. Given that about 60% of menopausal women seek treatment [3], improved management of hot flushes is an increasingly important public health issue [4].

The currently recommended [5] and also the most effective treatment is hormone therapy [6, 7]. For decades, it was the treatment of choice until the Women’s Health Initiative (WHI) study linked hormone therapy to higher risks of developing breast cancer, coronary heart disease, stroke, and endometrial cancer [8, 9]. Although the sweeping interpretation has been updated with refined recommendations dependent on individual risk factors, the WHI study nonetheless has been followed by a 79% drop in usage from 2002 to 2010 [10]. The challenge has since been to find non-hormonal alternatives in case of refusal or contraindication. Beneficial effects have been shown for the selective serotonin reuptake inhibitors gabapentin and clonidine [11, 12]. Herbal remedies are often used (45–63%) [13–15], although current evidence is mixed, with the overall trial quality rated to be suboptimal [16–19]. Acupuncture and relaxation techniques were found to be non-effective [20, 21], while cognitive-behavioral therapy, clinical hypnosis, and mindfulness-based stress reduction showed some positive results in alleviating hot flush-related distress [22–24].

Notably, placebo responses are high throughout hot flush trials [25, 26]. In studies of non-hormonal therapies, response rates range from 27% to 52% [27]. In estrogen trials, participants in the placebo arms obtain an averaged 58% reduction in hot flushes [6]. It is unclear whether these beneficial effects reflect a true placebo effect (i.e., a distinctive effect over and above natural history and regression to the mean) and, if so, whether these effects can be exploited to improve hot flush treatments.

It has long been viewed that placebos cannot be administered in accordance with ethical values since deception would be necessary for the occurrence of beneficial effects. This notion has been shaken up by studies on open-label placebos (OLP) showing that placebos can lead to positive effects even though their inert nature *is* disclosed [28–35]. The first randomized-controlled OLP trial demonstrated a significant improvement of irritable bowel syndrome after 3 weeks of placebo intake over an untreated control group with 60% (vs 35%) reporting adequate relief [30]. Clinical trials have since been conducted among patients with chronic low back pain [28], cancer-related fatigue [36], as well as smaller ones on depression [31] and allergic rhinitis [32, 33], with reporting of medium to large effect sizes. Experimental studies with healthy cohorts have also been conducted [29, 34, 35]. Up to now, the number of trials is small and underlying mechanisms are unclear.

The objective of this study is to determine whether OLP is efficacious to treat hot flushes. Further, we will analyze the response rate of OLP and health-related quality of life, whether beneficial effects can be maintained or increased with a prolonged placebo treatment, and whether beneficial effects are related to positive expectations.

## Methods/design

### Study design

**Fig. 1:**
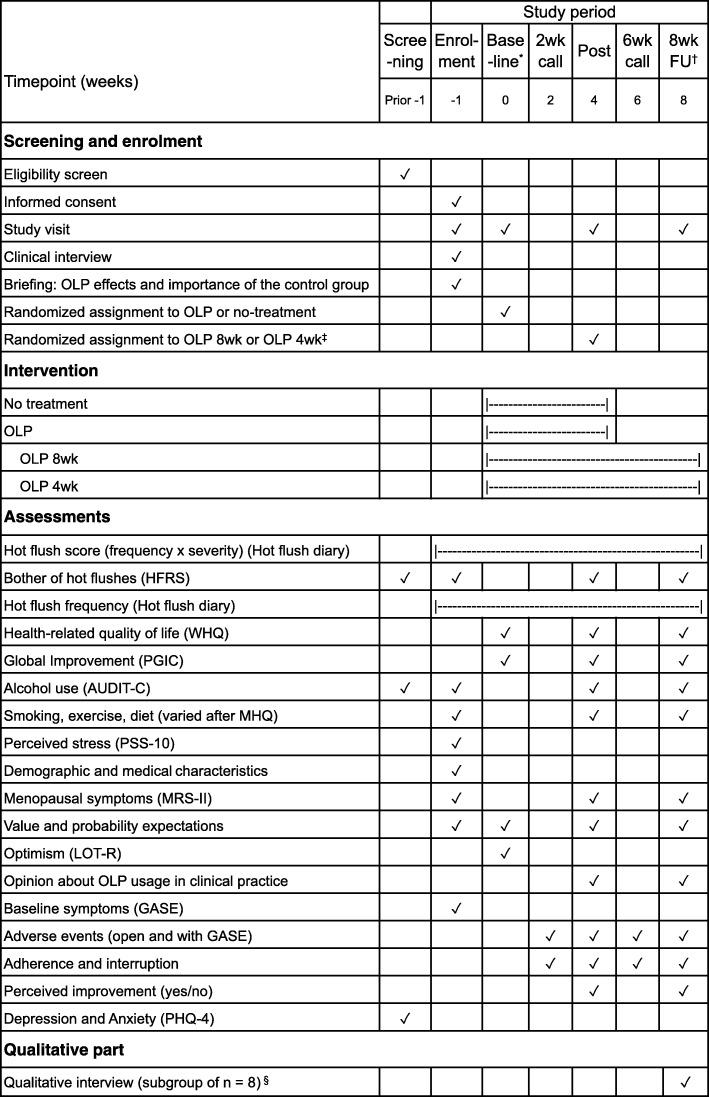
Schedule of enrolment, intervention, and assessment according to SPIRIT. *Post* post-treatment, *wk* week, *FU* follow-up, *OLP* open-label placebo, *HFRS* Hot Flush Rating Scale, *WHQ* Women’s Health Questionnaire, *PGIC* Patient Global Impression of Change, *MRS* Menopause Rating Scale, *MHQ* Menopause Health Questionnaire, *AUDIT-C* Alcohol Use Disorders Identification Test—Consumption, *PHQ* Patient Health Questionnaire, *PSS* Perceived Stress Scale, *LOT-R* Life Orientation Test—Revised, *GASE* Generic Assessment of Side Effects. Hot flushes are assessed ambulatory via the hot flush diary. *At baseline, health-related quality of life and expectations are assessed before and after the allocation, respectively. ^†^The fourth study visit and the 8-week follow-up assessment takes place for the OLP 8wk and OLP 4wk groups only. ^‡^For the second allocation, the OLP group is further divided into the OLP 8wk and the OLP 4wk groups. ^§^The interview is conducted at week 8 or later

In this randomized-controlled, assessor-blinded superiority trial, menopausal women with hot flushes are assigned 1:1 to a 4-week OLP treatment or a no-treatment control group. The control group allows for examining whether OLP has beneficial effects over and above spontaneous improvement and statistical phenomena. Patients receive three study visits at enrolment, baseline, and post-treatment (Fig. 1). Women allocated to OLP are randomized a second time at post-treatment to either another 4 weeks of placebo (OLP 8wk) or to discontinue the treatment (OLP 4wk). Accordingly, OLP 8wk and OLP 4wk receive a fourth study visit at 8 weeks follow-up, whereas, for the no-treatment group, the study ends at week 4. Two calls at weeks 2 and 6 are conducted to inquire about adverse events and adherence. To further understand the experiences of participants and potential psychological mechanisms from their perspective, qualitative interviews will be conducted with a subgroup of patients who have reported improvements under the placebo. Ethics approval is given by the Physicians’ Chamber of Hamburg (reference number PV5787). The trial is registered at ClinicalTrials.gov (ID NCT03838523) and complies with the SPIRIT guidelines [37].

### Study sample

Eligible women are required to (1) experience at least five moderate or severe hot flushes per day, including at night, (2) with at least moderate ratings of bothersomeness (sum score ≥ 16 on the bother subscale of the Hot Flush Rating Scale [38]), (3) be fluent in the German language, and (4) be in the menopausal transition (irregularities ≥ 60 days in the past year), or postmenopausal (cessation of menstruation ≥ 1 year) [39]. Exclusion criteria are use of hormonal therapy, herbal remedies to treat hot flushes, or intake of selective serotonin reuptake inhibitor (SSRI)/serotonin norepinephrine reuptake inhibitor (SNRI) within the last 6 weeks before enrolment, previous oophorectomy, severe physical or cognitive impairments which would constitute a barrier to give informed consent, severe depression or anxiety (≥ 9 sum score or ≥ 5 depression or anxiety subscore on the Patient Health Questionnaire (PHQ)-4) [40], and medical conditions which might cause hot flushes such as untreated hyperthyroidism, alcohol abuse (≥ 4 on AUDIT-C) [41] and cancer. After 8 weeks follow-up, four patients from each of the OLP 4wk and the OLP 8wk group who indicated symptom improvement are invited to take part in the qualitative interview (*n* = 8).

### Power analysis

The power calculation is based on our primary outcome hot flush score. The software G*Power was used to calculate the sample size a priori [42]. Since no OLP study has been conducted in the field of hot flushes, we base our sample size calculation on two separate clusters of information. From double-blind hot flush trials we can expect a moderate effect size of Cohen’s *d* = 0.40 given a mean of 19, standard deviations of 10 [23], and a four-point reduction in the hot flush score (frequency × severity) after 4 weeks of placebo [43]. Based on previous OLP trials in patients with irritable bowel syndrome and chronic low back pain, a large effect size of *d* = 0.80 can be expected [28, 30]. Combining these two clusters of information, a moderate to large effect size is expected for the difference between the OLP and the no-treatment groups at post-treatment. For a two-tailed Student’s *t*-test with an *α*-error rate of 0.05, a number of *N* = 90 participants would provide an 80% power to detect an effect of *d* = 0.60. We assumed an attrition rate of 10% and obtained our required sample size of *n* = 100 women.

### Setting

This trial is conducted at the Psychosomatic Institute and Outpatients Clinic of the University Medical Center Hamburg-Eppendorf in Hamburg, Germany. Women are recruited through physician referrals, advertisements in newspapers and the internet, and flyers distributed in the greater Hamburg area. The treatment is described as “novel mind-body treatment”. Interested volunteers can contact the study team for more information and, when positively screened on the phone, schedule the first study visit. Participants receive a reimbursement for their time to complete the hot flush diary and further questionnaires at the study visits.

### Procedures

Prior to enrolment, informed consent is obtained by all participants. The informed consent and all study visits are conducted by clinicians (MD or MSc psychologists). Self-report questionnaires are completed at each study visit, accompanied by a blinded assessor (study assistant). The assessor also gives instructions about how to complete the hot flush diary. Confidentiality is secured by replacing the participant’s identifying information with a number. All identifying information is stored separately from the data. The file which links the number to the participant is stored locally; access is only granted to study clinicians and the principal investigator (PI). The file is deleted after the publication of results.

Clinicians are encouraged to use active listening during consultations, to avoid technical jargon and to devote equal attention, empathy, and interaction time to all participants, irrespective of allocation. A semi-scripting of the visits ensures both consistency and naturalness of patient–clinician interactions. We expect participants to vary in terms of their wish to talk about symptoms, skepticism towards the treatment, etc. Hence, we allow interaction times to differ by up to 10 min for the first and up to 5 min for any other session. All information about the placebo treatment and the no-treatment group is provided prior to randomization and, hence, equally to all participants irrespective of group allocation. This ensures similar interactions with clinicians in both groups. Adverse events, including worsening of symptoms, are inquired about at each study visit/call to facilitate clinician support and, if necessary, initiate study discontinuation. All study visits (SV) and calls are outlined in the following.

### SV 1: Enrolment—clinical interview and placebo briefing

After informed consent is signed, a clinical interview inquiring about hot flushes (duration, severity, bothersomeness) and related medical characteristics is conducted (see measures). Then, all patients are given a placebo briefing before randomization: (1) The placebo effect is powerful; placebos given under uncertain conditions, i.e., in clinical trials, have been shown to produce significant alleviation of hot flushes. (2) Positive expectations might be helpful but are not necessary for the placebo to be effective. (3) The underlying mechanisms of OLP are unclear. Conditioning processes could constitute one of them. That is, the body might react to placebo pills in an automatic way since it has learned to associate the pill intake with symptom improvement. (4) Disbelief or doubts are fine but taking the pills faithfully is essential for the generation of a positive effect. Finally, (5) no OLP study has yet been conducted in the field of hot flushes. Hence, we encourage patients—if allocated to OLP in the following week—to “wait and see what will happen”. Placebos are described as pills without pharmacologically active substances. To minimize stigmatizing associations of placebos (e.g., only gullible people may benefit from placebos), we inform patients that placebos have been shown to produce measurable biochemical changes in the body. A short discussion about the importance of the no-treatment group follows. The session is concluded by informing the patient about further procedures. The first study visit takes about 30 to 40 min (time for questionnaire completion not included).

### SV 2: Baseline—allocation

At the second study visit, patients are informed about their assignment by a clinician. Clinicians are not aware of the group assignment until the patient has opened the envelope and disclosed the allocation result. Clinicians instruct the OLP group to take two pills a day for 4 weeks, each morning and evening after a meal. A total number of 56 pills are handed out in paper packaging which includes the pill bottle and the original medication leaflet of the producing company. The bottle shows the name of the pill (“placebo”), the number of pills and its equivalent in grams, a subtitle (“for menopausal hot flushes”), the contact data of the responsible party (“Department of Psychosomatic Medicine and Psychotherapy”) and further instructions regarding the pills (e.g. “Store inaccessible to children”). Women are reminded about the importance of taking the pills faithfully and a 50% chance of continuing the treatment for another 4 weeks. Women assigned to the no-treatment group are reminded of the meaning of this study group. The duration of the second study visit is 5 to 10 min.

### Two-week call

The clinician asks about the occurrence of adverse events, including aggravation of hot flushes. The OLP group is additionally asked about treatment adherence and whether treatment has been interrupted.

### SV 3: Post-treatment—second allocation (OLP group) and study conclusion (no-treatment group)

At the third study visit, all patients are asked about their hot flushes and adverse events by the clinician. Patients in the OLP group are then assigned for a second time; assignment results are disclosed by the clinician. Patients in both OLP 4wk and OLP 8wk groups are asked to complete the diary for another 4 weeks. The OLP 8wk group receives another bottle of placebos. For the no-treatment group, this constitutes the last study visit. Study diaries are collected and women are thanked for their participation. The third study visit takes 10 to 15 min.

### Six-week call (OLP 4wk and OLP 8wk)

The same questions as in the 2-week call are asked in this 6-week call.

### SV 4: 8-week follow-up—study conclusion (OLP 4wk and OLP 8wk)

Patients of the OLP 4wk and the OLP 8wk groups are asked about their hot flushes and adverse events by the clinician. Study diaries are collected and women are thanked for their participation. The fourth study visit takes 10 to 15 min.

### Qualitative interviews (subgroup of *n* = 8)

Shortly after the fourth study visit, a subgroup of participants is interviewed about their overall experiences with the treatment. The interview will be set up and analyzed with the interpretative phenomenological analysis (IPA) method, which facilitates the understanding of subjective experiences and favors an in-depth exploration by using a loose agenda over a large number of participants [44]. In alignment with the recommendations of the method, we aim to include a rather homogeneous sample. Thus, we will include only women who undergo the placebo treatment *and* indicate a perceived improvement, either at post-treatment or at 8-week follow-up. Of those patients, each one has a 50% chance to be invited to the interview, which is decided by drawing a card (yes/no). Informed consent is obtained separately from the quantitative part of the study, right before the scheduled interview. Ongoing enrolment is conducted until the full sample is reached. Themes include (1) how hot flushes affected everyday life before study participation, (2) prior experiences with hot flush treatments, (3) study motivation, including expectations and hope regarding the treatment, (4) subjective explanation for improvement, and (5) symptom perception or change in symptom perception. The interview is recorded and transcribed verbatim.

### Blinding

Due to the nature of the study, patients and clinicians are aware of group assignment. Assessors are blinded to group assignment of participants. To prevent the breaking of blinding, participants are requested to not communicate their group affiliation when they have questions about the assessments.

### Randomization

Two randomizations are conducted. The first randomization takes place at baseline (second study visit) for all patients, and the second one takes place at post-treatment (third study visit) for the OLP group only. Both randomization sequences are generated prior to the first enrolment using an online program (Sealed Envelope). A researcher who is otherwise not involved in the study notes the results of the allocation sequence and uses opaque, sealed, and sequentially numbered envelopes for its concealment. The sequences are then saved and locked away by the principal investigator (YN). Hence, it is not accessible to any person involved in the conduct of the immediate study. The allocation lists are stored in an office room of the department, inaccessible to blinded assessors throughout the study.

For the first randomization in which patients are 1:1 assigned to either OLP or no-treatment, we use permuted block randomization. The assignment is performed by the clinician at the beginning of the second study visit by opening the envelope.

For the second randomization, we use stratified permuted block randomization. Patients in the OLP group will be randomized 1:1 to OLP 4wk and OLP 8wk. The assignment is stratified for perceived improvement of hot flushes (yes/no). The clinician determines the stratum based on the patient's statement and then performs the allocation.

### Measures

#### Primary outcomes

The group difference between OLP and no-treatment in the hot flush composite score (frequency × severity) and bothersomeness of hot flushes at post-treatment are our primary outcomes. Women record number and severity of hot flushes in real-time, i.e., at occurrence, using a portable paper diary [25, 45]. Night sweats are recorded on subsequent mornings. The diary is the gold standard for assessing hot flushes [46] and has shown high reliability and validity [25]. In accordance with guideline recommendations, the severity of each hot flush is rated as mild, moderate, or severe [5]. The severity categories are predefined, which minimizes divergence due to subjective evaluations. In the day, mild hot flushes are *not* accompanied by sweating. Moderate hot flushes are accompanied by sweating, whereas severe hot flushes lead to disruption of current activity. At night, mild hot flushes are spotted by damp sheets/clothing, whereas moderate and severe hot flushes cause awakening. Severe hot flushes additionally necessitate actions like opening the window, removing sheets, etc. The Hot Flush Rating Scale [38] assesses bothersomeness, i.e., to what extent hot flushes are regarded as problematic and distressing and cause interference with daily life in the preceding week. The Hot Flush Rating Scale has good reliability and validity [38].

#### Secondary outcomes

Secondary outcomes include the group difference between OLP and no-treatment in hot flush frequency, health-related quality of life, global improvement, and the number of responders (≥ 50% in hot flush frequency at 4 weeks from baseline) at post-treatment [47]. Hot flush frequency is assessed as part of the diary. Health-related quality of life is measured with the Women’s Health Questionnaire (WHQ) [48], a questionnaire of proven reliability and validity [49] which covers eight domains including depressed mood, somatic symptoms, memory/concentration, vasomotor symptoms, anxiety, sexual life, sleep, and attractiveness. Although part of the questionnaire, the menstrual symptoms scale is excluded from analyses since many patients would be post-menopausal. Patients indicate their global improvement of hot flushes on a seven-point Likert scale (1 ‘very much worse’, 2 much worse’, 3 ‘minimally worse’, 4 ‘no change’, 5 ‘minimally improved’, 6 ‘much improved’, 7 ‘very much improved’) of the Patient Global Impression of Change scales (PGIC) [50]. The PGIC is a validated and commonly used questionnaire in the field of pain and has been previously used in a sample with menopausal women [51].

#### Medical and sociodemographic characteristics, hot flush-related lifestyle, and psychosocial variables

Medical characteristics are assessed via the clinical interview and include menopausal transition state, hysterectomy, years of hot flushes, previous intake of hot flush medication including experiences of benefits and adverse events, current body-mind interventions against hot flushes, and baseline symptoms. Psychotherapy, body mass index, and menopausal symptoms (Menopause Rating Scale-II) [52] are assessed via self-report questionnaires. Demographic variables include age, marital and occupational status, and education level. Lifestyle variables include smoking, exercise (modified after the Menopause Health Questionnaire) [53], and alcohol consumption (AUDIT-C) [41]. Perceived stress (PSS-10) [54, 55] was shown to be associated with frequency, severity, and duration of hot flushes and might thus be a potential cofounder of the treatment effect [2, 56, 57].

#### Expectations

Participants’ (1) value expectations or hopes/wishes/desires about the treatment, (2) probability expectations, and (3) dispositional expectations, i.e., optimism, are assessed. Due to the lack of validated questionnaires in the field of expectations [58], we created two items (value expectations: “What change would you like to happen to your hot flushes over the next 4 weeks?”; probability expectations: “How do you expect your hot flushes to change over the next 6 weeks?”) which are rated on a scale from 0 (*no change*) to 10 (*maximum improvement*). These items are based on a qualitative interview study with lower back pain patients [59]. We assess optimism with the Life Orientation Test-Revised (LOT-R) [60]. Optimism might constitute a predictor of placebo response, although evidence remains mixed [61, 62].

#### Adverse events

Adverse events are assessed openly and using a questionnaire. The list of symptoms in the self-report questionnaire includes loss of appetite, dry mouth, sleeping problems, nausea, dizziness, fatigue, constipation, nervousness, mood changes, and blank spaces for the specification of further symptoms. Severity (0 = not present, 1 = mild, 2 = moderate, 3 = severe) is rated for each symptom within the validated format of the GASE scale [63]. To discern whether adverse events may be related to the treatment, the same list of symptoms is assessed at baseline.

#### Adherence and treatment interruption

Intake-related information is assessed at weeks 2, 4, 6, and 8. Adherence is assessed via self-report with a single item (“How many placebo pills have you actually taken during the last week?”), which has been validated in a previous study with breast cancer patients [64]. Treatment interruption is assessed through open-ended questions (“Did you interrupt the treatment? If yes, for how many days?”).

#### Opinion about OLP usage

The question “Do you think OLP prescription in clinical practice is acceptable?” has been asked in a previous telephone survey study and is to be answered on a scale from 1 (‘definitely yes’) to 4 (‘definitely not’) [65].

#### Perceived improvement

The item “Have your hot flushes improved in the last 4 weeks?” (yes/no) is used as a stratum for the second randomization and as an indicator (“yes” as a requirement) for the qualitative interview.

### Data management

All data collection procedures have been made semi-manual. To reduce errors in data entry, every item answer is labeled with the original questionnaire score. After data entry, 10% of the data is controlled. If the number of mistakes exceeds 5% per case, another 10% of the data is controlled, etc. Plausibility checks are conducted before the statistical analyses.

### Statistical analyses

All hypotheses are tested two-sided with an *α*-level of 0.05.

#### Missing data

**Fig. 2:**
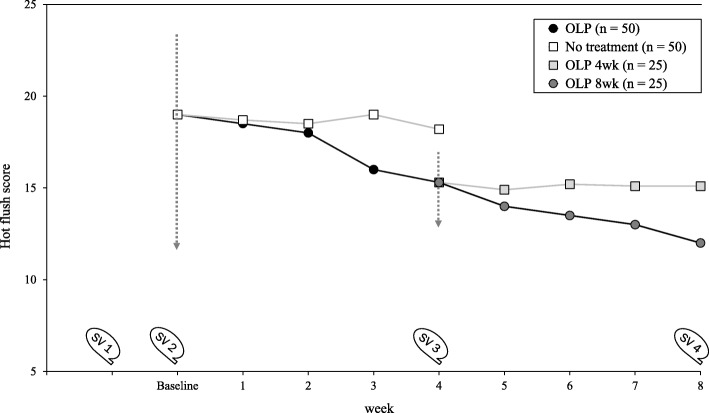
Study design with hypothesized trends of hot flush score. *SV* study visit, *OLP* open-label placebo, *wk* week. The *arrows* represent the point in time of the first and second allocation. *Circles* represent placebo intake, *squares* represent no placebo intake. OLP 4wk and OLP 8wk are subgroups of the OLP group

To analyze diary data, we aggregate daily data so that each data point comprises a weekly mean (Fig. 2). Means are calculated if at least 3 days of the week are completed. Almost identical means of the hot flush diary were found for 3 and 7 days [66]. Missing data points are not replaced since, in linear mixed models, missing outcome data are handled using maximum likelihood estimation, assuming that data are missing at random conditional on information in the model. Single missing values in questionnaires are substituted by the mean of the remaining items, provided that 80% are completed [67].

#### Efficacy

For our primary outcome hot flush composite score, we conduct a linear mixed model for repeated measures using restricted maximum likelihood estimations with the intent-to-treat sample. We define group membership (OLP/no treatment), measurement point, and their interaction term as fixed effects. The baseline score is additionally included as a covariate. Measurement points are treated as repeated measures with an autoregressive residual covariance structure. Moreover, the model includes a random intercept to model between-subject differences. A normal distribution with an identity link is used. Since each data point reflects a weekly mean, up to five weekly means are included for each participant. Effect sizes are obtained by dividing the estimated group difference by the product of standard error by square root of the number of participants in the no-treatment group [68]. For our primary outcome bothersomeness of hot flushes, we will perform a linear mixed model with two data points at baseline and post-treatment. A post-hoc test comparing OLP with no-treatment will be conducted to test the group difference at post-treatment.

By fitting (generalized) linear mixed models, the analytical strategy for all secondary outcomes corresponds with the one described for the primary outcome. For metric outcomes (health-related quality of life and global improvement) we use a normal distribution with an identity link. For dichotomous outcomes (response), we use a binomial distribution with a logit link, and for count outcomes (hot flush frequency), we use a negative binomial distribution with a log-link.

#### Sensitivity analyses

Sensitivity analyses include rerun of outcome analyses with missing values substituted using the last-observation-carried-forward method, per-protocol analyses (exclusion of patients who have discontinued treatment or reported adherence rates < 90%), analyses with adjustment for the variables ‘years of hot flushes’, and stress, and after exclusion of patients who have conducted lifestyle changes in the course of the study.

### Further research questions

#### Duration

To investigate whether a prolonged intake facilitates increased symptom improvements, linear mixed models which align with the ones of the efficacy question are performed to examine differences between the groups OLP 4wk and OLP 8wk. With *n* = 25 participants per group, significant effects would be present if the effect size was Cohen’s *d* = 0.8. Since no study has investigated the duration of OLP in a randomized-controlled design, we are unaware of whether an effect exists at all. Hence, the comparison between the two OLP groups is exploratory and the study not deliberately powered for this sub-analysis.

#### Maintenance

To test whether beneficial effects can be sustained after the intake is stopped, we perform contrast analyses within the OLP 4wk group with the pre-defined contrasts (A) ‘baseline’ vs ‘4 wk’, (B) ‘baseline vs ‘8 wk’, and (C) ‘4 wk’ vs ‘8 wk’. If sustained efficacy is given, contrasts (A) and (B) would be statistically significant, whereas contrast (C) would be non-significant. Aligned with the “duration” question, this question is also an exploratory one.

#### Adverse events

To investigate treatment safety, we will perform an exploratory comparison of rates of adverse events between the OLP group and the no-treatment group. Individual baseline symptoms will be considered when interpreting the results.

#### Role of expectations

To investigate whether positive expectations amplify beneficial effects of the OLP treatment, value expectations, probability expectations, and optimism are separately included in the primary outcome models as candidate moderators. The interaction effect time × group × expectation variable will be tested with significant effects indicating that expectation moderates group effects. The trajectory of value and probability expectations over time will also be subject to exploratory analysis.

#### Qualitative analysis

According to IPA procedures, transcripts are analyzed line-by-line to find emerging themes of each participant. Themes are then clustered within patients and compared between patients. The first two interviews will be transcribed and analyzed soon after execution by two researchers. Notes and themes are compared and discrepancies discussed. Further interviews are analyzed by the researchers independently.

## Discussion

This trial is the first to investigate the efficacy of non-concealed placebos for hot flushes among menopausal women. In a randomized-controlled design, we compare 4 weeks of twice daily OLP intake with no treatment. By subsequently dividing the treatment group into two subgroups (continuing vs discontinuing treatment), we will explore the question of how long OLP effects last and whether beneficial effects can be maintained after treatment is discontinued. Additionally, subjective experiences with the placebo treatment are examined by qualitative analysis of participant interviews. Our aim is to uncover whether beneficial effects of double-blind placebos in hot flush trials may also apply to their open-label surrogate [69].

Presumably, the biggest question for OLP is why it works. In the tradition of placebo research, expectations, conditioning, and the patient–practitioner relationship have been suggested as underlying mechanisms [70, 71]. Expectations, i.e., beliefs about future outcomes, are the only potential mechanisms which have been examined as part of OLP studies. The positively framed rationale about placebos may act as a verbal suggestion which enhances positive expectations. One study showed that OLP administration with or without the briefing did not result in differing allergic symptoms [33], whereas another study with healthy participants did find divergent analgesic effects depending on the briefing [34]. Interestingly, in the latter study, expected pain did not differ between the groups. We also assess expectations in both groups to compare whether they differ after allocation and whether they predict the outcome.

Conditioning is based on prior treatment experiences and occurs when neutral stimuli and active treatment are repeatedly paired (associative learning). Since OLP studies did not incorporate conditioning paradigms, the magnitude of conditioning and whether it plays a role cannot be determined. Previous studies included clinical samples which, based on results of qualitative studies, most probably experienced treatment disappointments which would render the conditioning hypothesis contradictory to the beneficial effects of OLP [71].

The patient–practitioner relationship is an important contextual factor which has a small but significant effect on outcomes across diseases [72]. Since patient–practitioner interactions in OLP studies are warm and attentive [28, 30], it may contribute to OLP effects. However, since both groups are kept “constant” considering the relationship, it is unlikely that OLP effects are explicable by the relationship only [71].

An often discussed model for placebo effects is the predictive coding model [73–75]. According to this approach, the brain does not simply process incoming, bottom-up signals. So-called priors, i.e., predictions generated by the brain, affect perception directly, thus resulting in diverging ‘real-world’ perceptions between people. Those predictions are based on the “integration of sensory inputs, prior experience, and contextual cues” (p.1 in [75]). In analogy to Bayesian models, the brain aims to minimize the mismatch between descending priors and ascending signals. If a prediction error occurs, priors are changed accordingly, thus allowing for effective navigation through the world. Notably, priors and signals can take on different degrees of precision. It is argued that strong priors with high precision in combination with vague signals can lead to perceptions corresponding with the priors [74]. Kirsch [76] suggests that given most bodily signals in chronic disease are ambiguous, priors tend to act as self-fulfilling prophecies with regard to symptoms. Importantly, they may or may not be consciously accessible. The role of nonconscious processes is corroborated by recent experiments of Jensen et al. showing that placebo effects can be both acquired (with conditioning) and triggered using subliminal cues (for review, see [77]). Consciously accessible priors would constitute expectations [78].

As pointed out by Wiech [79], having utmost strong and precise (positive) priors might be detrimental since it creates a divergence between prediction and input which undermines the function of minimizing error. Kaptchuk [71] has put forward that, in chronic conditions that fluctuate over time, such as hot flashes, highly precise and strong priors would lead the brain to interpret variations as being just more hot flashes. OLP efficacy may involve introducing its own form of therapeutic uncertainty that shakes up those priors. In previous OLP studies, participants were not simply confident about symptom improvement under placebo. This finding aligns with the results of qualitative studies. For example, patients undergoing acupuncture treatment [80], or blind placebo treatment [81], reported not wanting to expect too much. Subsumed under “the paradox of hope”, this framework describes a balancing act between having enough hope to undergo potentially helpful treatment but refraining from overly positive expectations in order to avoid disappointment [82]. With placebos being widely acknowledged as non-efficacious, patients could preserve some skepticism towards OLP, even when a rationale is provided. However, the uncertainty—in combination with the hope for improvement or desire of relief as it is named in other studies [83]—may generate imprecise but positively toned priors which might have facilitated perception in favor of those priors [70].

Due to the small number of trials, we have confined ourselves to an overview of the proposed mechanisms. Unlike the traditional picture, it is likely that these mechanisms are not mutually exclusive but rather comprise an integrative framework. Further studies are warranted to examine the aforementioned mechanism hypotheses.

### Limitations

There are several limitations and unaddressed questions in our study. One of these is whether OLP generates physiological changes. Two recent experimental OLP studies on healthy people found no effect of OLP on objective parameters [35, 84]. Also, placebo effects in clinical trials across diseases [85], as well as specifically in hot flush trials [86], are known to be caused or amplified by a subjective reporting bias. The physiological measurement of hot flushes is compounded by the circumstance that concordance rates between self-rated and physiological measures are low [47, 87], which renders an interpretation of its dissonance difficult. Although limitations are present, the ambulatory assessment of symptoms minimizes potential biases like memory and emotions. The diary is also the gold standard for hot flush assessments, which allows for comparison with other studies.

Another limitation is a possible self-selection bias. In postings, we will describe our treatment as a novel “mind-body treatment”. This might attract women who are particularly open to alternative and complementary treatments. However, as discussed by Kaptchuk et al. [30] and Kelley et al. [31], self-selection also occurs in clinical practice, i.e., patients are given recommendations by the practitioner and thereby also the choice of accepting or declining treatments. The previous use of hormonal or non-hormonal treatments is assessed in this study to further explore this discussion point.

### Relevance

The findings of this trial aim to contribute to the following objectives: providing evidence-based treatment options for menopausal women, exploring non-specific effects in the context of hot flush treatment, and adding to the list of OLP studies.

OLP effects might manifest for those conditions with high baseline variability and large effects under placebos in double-blind trials [70]. However, the clinical indication of OLP is currently indefinite. Ballou et al. [88] and Carvalho et al. [28] discussed that OLP could be prescribed additionally to a “watch-and-wait” strategy. This suggestion matches up with studies about practitioners’ perspectives on placebo prescription. These have consistently found that placebos are frequently administered when treatments are expected or demanded [89]. Placebos with consent could thus constitute the solution to this humane dilemma of complying with guidelines and respecting patients’ wishes [90]. Another possibility is its application as a “dose extender” of another treatment [91]. After repeated pairing with an active treatment, placebos could be used to partly substitute the active treatment. The up to now only study of dose-extending OLP has been conducted with children diagnosed with attention deficit hyperactivity disorder (ADHD), which enabled the same benefits while reducing adverse events. Supplementary interviews indicated a high acceptance of OLP among parents [92]. Comparably, a telephone survey study in the general population has found that over 50% of the participants regarded OLP administration as ethical and would consider using OLP if recommended by their physician [66]. The exploratory questions about duration and maintenance of OLP effects, which this trial additionally aims to answer, are of special relevance to these potential clinical applications of OLP.

Our second objective is grounded in the finding that, in most clinical hot flush trials, complementary and alternative medicine (CAM) show no benefit over and above placebos [18, 93]. Usage of CAM is highly prevalent [15], yet not warranted given its inconclusive evidence [16, 17]. Beneficial effects of CAM and double-blind placebo may reflect non-specific effects or simply natural history or statistical phenomena. If the OLP arm demonstrates improvements over the no-treatment group, this might indicate that non-specific effects are strong. This information, again, can be incorporated when designing future trials by minimizing or systematically controlling for non-specific effects [94]. Vice versa, these non-specific effects might be systematically exploited to maximize treatment effects.

Since OLP administration as conducted in clinical studies includes transparent disclosure of its use, it complies with ethical standards [95]. Therefore, in cases of a personal decision against hormonal therapy, SSRIs or SNRIs, gabapentin or clonidine, and in light of the unclear evidence of CAM, open-label placebos might constitute a viable option to treat menopausal hot flushes in the future.

## Trial status

Protocol version number 1 from May 22nd, 2018. Recruitment started on October 1st, 2018 and is currently ongoing.

## Data Availability

Not applicable.

## Abbreviations

ADHD: Attention deficit hyperactivity disorder
AUDIT-C: Alcohol Use Disorders Identification Test—Consumption
BMI: Body mass index
CAM: Complementary and alternative medicine
GASE: Generic assessment of side effects
HFRS: Hot Flush Rating Scale
IPA: Interpretative phenomenological analysis
LOT-R: Life Orientation Test—Revised
MHQ: Menopause Health Questionnaire
MRS: Menopause Rating Scale
OLP: Open-label placebo
PGIC: Patient Global Impression of Change
PHQ: Patient Health Questionnaire
PI: Principal investigator
PSS: Perceived Stress Scale
SNRI: Serotonin norepinephrine reuptake inhibitor
SSRI: Selective serotonin reuptake inhibitor
SV: Study visit
WHQ: Women’s Health Questionnaire
